# Extraction, Characterization, and Antioxidant Activity of *Eucommia ulmoides* Polysaccharides

**DOI:** 10.3390/molecules29204793

**Published:** 2024-10-10

**Authors:** Yuqing Peng, Yulin Yang, Yitong Tian, Meng Zhang, Kang Cheng, Xuelei Zhang, Mengqing Zhou, Ming Hui, Yong Zhang

**Affiliations:** College of Biological Engineering, Henan University of Technology, Zhengzhou 450001, China; 2022930654@stu.haut.edu.cn (Y.P.); yyl980330@163.com (Y.Y.); snowcopper@163.com (Y.T.); 221060500422@stu.haut.edu.cn (M.Z.); chengkang68@126.com (K.C.); zhangxuelei1020@126.com (X.Z.); zhoumq2005@163.com (M.Z.); huiming@haut.edu.cn (M.H.)

**Keywords:** *Eucommia* polysaccharide, extract, antioxidant

## Abstract

Herein, the ultrasound-assisted extraction conditions affecting the yield of EUPS (*Eucommia ulmoides* polysaccharide) were analyzed using a Box-Behnken response surface design. The alleviation effect of EUPS on diquat-induced oxidative stress in mice was also studied. A maximum EUPS yield of 2.60% was obtained under the following optimized conditions: an extraction temperature of 63 °C, extraction time of 1 h, and ratio of liquid to raw materials of 22:1. EUPS exhibited strong 1,1-diphenyl-2-picryl-hydrazyl (DPPH) radical-scavenging ability (87.05%), 2′-Azinobis-(3-ethylbenzthiazoline-6-sulphonate) (ABTS) radical-scavenging ability (101.17%), and hydroxyl radical-scavenging ability (62.92%). The administration of EUPS increased the activities of superoxide dismutase, catalase, and glutathione peroxidase and decreased malondialdehyde levels in the livers of mice exposed to diquat. EUPS may inhibit the downregulation of NAD(P)H:quinoneoxidoreductase 1 and heme oxygenase 1 mRNA expression in the livers of diquat-administered mice through the Nrf2-Keap1 signaling pathway. Moreover, the abundance of Firmicutes and *Ligilactobacillus* was enhanced, whereas that of Helicobacter decreased in the gut of the remaining groups of mice compared with that of the diquat-treated mice. Therefore, EUPS exhibited an antioxidant effect and improved oxidative stress and intestinal flora abundance in mice.

## 1. Introduction

*E. ulmoides* (*Eucommia ulmoides*), also known as bakelite, comes from the Eucommia family plant [1]. It is a valuable homologous resource with good medicinal value and edible benefits in China [2]. *E. ulmoides* has been officially recognized as a medicinal plant and is listed in the Chinese Pharmacopoeia [3]. In China, it is mainly distributed in Shanxi, Gansu, Zhejiang, Henan, Hubei, Sichuan, Guizhou, Yunnan, and other places [4,5]. Recently, the epidermis, male flowers, and seeds of *E. ulmoides* have been extensively studied and utilized in terms of medicinal value; however, the leaves, which are the most productive and valuable parts, have not been fully utilized. The antioxidant activities of *E. ulmoides* leaves have been previously reported [6,7]. *E. ulmoides* leaves contain several active ingredients, such as iridoids, phenols, flavonoids, lignans, sterols, and gutta-percha [8,9]. These active substances endow *E. ulmoides* with its antibacterial, anti-inflammatory, antioxidant, antitumor, and other beneficial properties [10,11]. As one of the main active components of *E. ulmoides* leaves, EUPS from *E. ulmoides* have attracted considerable attention because of their antioxidant, antitumor, antimicrobial, and immunomodulatory functions and are ideal candidates for food and biomedical industries [12,13].

Oxidative stress refers to the cellular damage and pathological changes that occur when there are more oxidants than antioxidants in an organism. When the liver and kidneys are subjected to oxidative stress, various antioxidant-specific gene expression and signal transduction pathways are activated to maintain homeostasis and protect the organs from oxidative damage. Antioxidant enzymes such as superoxide dismutase (SOD), catalase (CAT), and glutathione peroxidase (GSH-Px) are the first line of defense against oxidative stress in cells. They convert reactive oxygen species (ROS) into nontoxic compounds to reduce the damage caused by oxidative stress to the body. Gut microbes provide a degree of protection against oxidative stress-related diseases.

EUPS is a kind of heteropolysaccharide [14] which can significantly reduce serum alanine aminotransferase, aspartate aminotransferase, high mobility group protein B1, IL-1β, TNF-α, and malondialdehyde levels, and increase SOD activity [15]. Its antioxidant mechanism may also be related to the inhibition of TLR4/NF-κB pathway activation [16]; EUPS can also improve antioxidant capacity by reducing intracellular ROS production and mitochondrial membrane damage [17,18].

With the continuous discovery of various biological activities of *E. ulmoides* extract, more attention has been paid to *E. ulmoides* [19]. This study was conducted to extract EUPS and optimize the ultrasonic extraction of *E. ulmoides.* In addition, the purity, molecular weight, sugar content, and monosaccharide composition of EUPS were analyzed, and the antioxidant-scavenging activity of EUPS from *E. ulmoides* was determined via in vitro antioxidant assay. Moreover, the antioxidant activity of EUPS in mice was investigated. This study offers a theoretical basis for the development of EUPS.

## 2. Results and Discussion

### 2.1. Single-Factor Test

**Figure 1:**
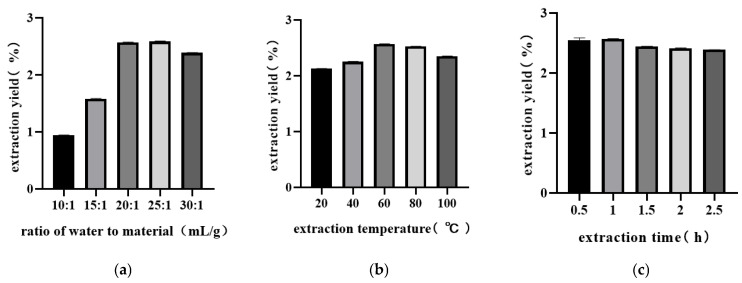
Effect of ratio of water to material (**a**), extraction temperature (**b**), and extraction time (**c**) on extraction yield of EUPS.

The effect of the ratio of water to raw materials on the extraction yield is shown in Figure 1a. With an increase in the ratio of water to materials from 10:1 to 25:1, the extraction yield increased rapidly. When the ratio of water to materials was 20:1, the extraction yield reached 2.60%. After that, the extraction yields gradually decreased. This may have occurred because increasing the solid-liquid ratio can help dissolve more polysaccharides from the sample by increasing the diffusion rate of the solvent in the cell. However, there is a limit to this effect, and when the solid—liquid ratio exceeds 20:1, the yield of the polysaccharides does not increase significantly and may decrease. This could be due to the weakening of the mechanical vibration effect of ultrasonic waves, which can decrease the yield of polysaccharides. This may also have happened because when the solid-liquid ratio was low, the sample was not sufficiently mixed with water and the extraction was incomplete, which is not conducive for the release of intracellular polysaccharides, resulting in a low extraction rate. When the ratio of materials to solvent was high, the extraction rate decreased, which might be because an increase in the solvent made the distance of internal diffusion longer, decreasing hydration and affecting the mass transfer. Figure 1b shows the effect of extraction temperature on the extraction yield; when the extraction temperature increased from 20 °C to 60 °C, the extraction yield increased from 2.13% to 2.58%. After that, the extraction yields gradually decreased. The movement of polysaccharides and their release and diffusion in the leaf cells of *E. ulmoides* can be accelerated by increasing the temperature within a certain range. However, if the temperature is too high, the polysaccharide might be degraded [20]. These findings are consistent with the fractionation of natural polysaccharides from Zizyphus jujuba cv. Jinsixiaozao [21]. As shown in Figure 1c, when the extraction time increased from 0.5 to 1 h, the extraction yield increased from 2.53% to 2.58%; after 1 h, the extraction yield significantly decreased. The possible reason is that the infiltration of solvent molecules and the dissolution of EUPS require a certain extraction time, but if the extraction time is too long, the structure of EUPS will be damaged by ultrasonic waves, thereby affecting the overall extraction rate of EUPS.

### 2.2. Fitting the Process Model

The effects of three individual variables, including the ratio of water to raw materials, extraction temperature, and extraction time, on the yields of EUPS in the factorial design are presented in Table 1. By applying a multiple regression analysis to the experimental data, the response and test variables were obtained using the following second-order polynomial equation:

R = −1.25225 + 0.14955A + 0.057963B + 0.70400C − 1.000000 × 10^−4^AC − 7.000000 × 10^−6^AC + 2.50000 × 10^−4^ × B × C − 3.42000 × 10^−3^A2 − 3.42000 × 10^−4^B2 − 0.39200C2

The experimental results were analyzed using ANOVA for extraction yield, and are cited in Table 2. The results show that among the test variables used in this study, A (ratio of water to material), B (extraction temperature), and A^2^, B^2^, and C^2^ were extremely significant for the extraction rate of EUPS (*p* < 0.01). However, C (extraction time) was significant (*p* < 0.05). AB, AC, and BC had no significant effect on the yield (*p* > 0.05).

### 2.3. Verification of Results

**Figure 2:**
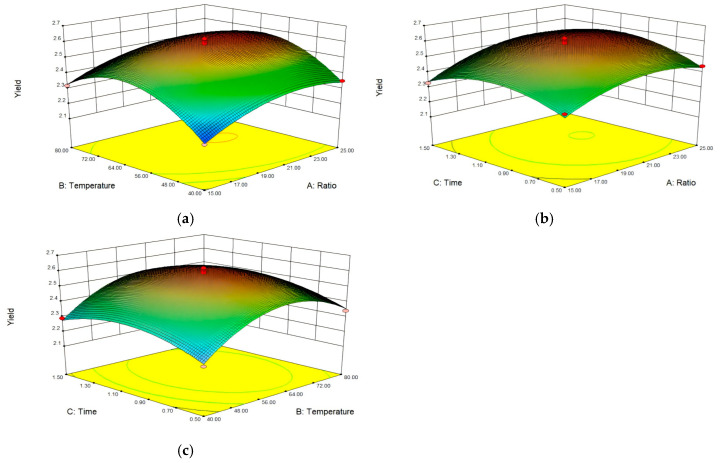
Three-dimensional surface plots showing the interaction effects of (**a**) extraction temperature and ratio of water to materials, (**b**) extraction time and ratio of water to materials, and (**c**) extraction time and extraction temperature on the extracted yield of EUPS.

Figure 2a–c shows contour and surface plots for the extraction percentage in the corresponding interaction effects. All three variables exhibited a significant effect on the extraction yields of EUPS.

Figure 2a shows the effects of extraction temperature and the ratio of water to materials on the extraction yields when the extraction time was at zero. This indicates that extraction temperature and time showed reciprocal interaction with yields. When the extraction temperature was fixed, the yields initially increased and then flattened with increasing extraction temperature. When the ratio of water to material was fixed, the yields initially increased and then decreased with increasing extraction temperature. As shown in Figure 2b, the extraction time and ratio of water to materials had a similar positive effect on the yields when the extraction temperature was at zero. Figure 2c shows that the extraction temperature and time exhibited a similar positive effect on the yields when the ratio of water to materials was at zero.

The ratio of water to raw materials exhibited the highest impact on extraction yield, followed by extraction temperature and time. Based on contour plots and variance analysis, the optimum operational conditions for maximizing extraction yield were a water:material ratio of 22:1, extraction temperature of 63 °C, and an extraction time of 1 h. Under the optimum operating conditions, a 2.60% extraction yield was achieved.

### 2.4. Characterization of EUPS

#### 2.4.1. Physicochemical Property Analysis of EUPS

EUPS is a milky white powder, which is soluble in water and insoluble in organic reagents such as ethanol and petroleum ether. The results of Fehling’s, iodination, and FeCl_3_ tests confirmed that the EUPSs comprised polysaccharides, but did not contain reducing sugar, polyphenol, or starch. The results of the Coomassie brilliant blue staining test indicated that it was almost free of proteins.

#### 2.4.2. Determination of Uronic Acid Content in EUPS

The uronic acid content of EUPS was calculated using the standard curve formula (y = 4.3129x + 0.6289, R^2^ = 0.9925), and the uronic acid content in EUPS was 13.8%. In previous studies, the monosaccharide composition of polysaccharides after acid hydrolysis was determined by analyzing and identifying the polysaccharides in *E. ulmoides*. There was a certain amount of uronic acid, and the content was 11.1–13.3% [22]. This is consistent with the results of this study.

#### 2.4.3. FT-IR Analysis of EUPS

**Figure 3:**
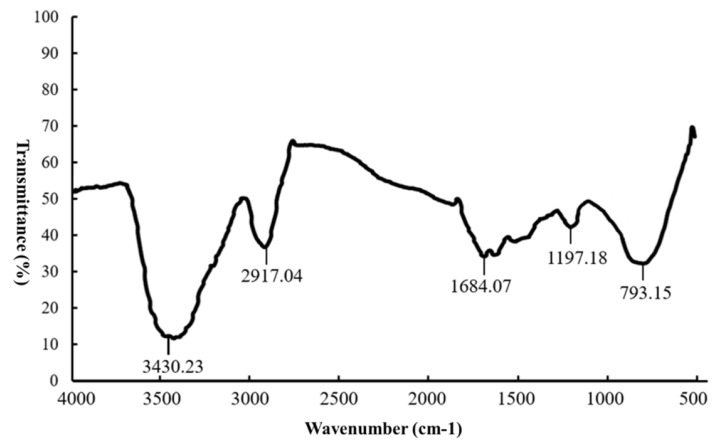
FI-IR spectra of EUPS.

The infrared spectrum of the extracted EUPS is shown in Figure 3. There was a strong and wide absorption peak at 3430.23 cm^−1^, which is related to the stretching vibration of the –OH. A peak value of 2917.04 cm^−1^ was found for the angular vibration at C–H, 1683.94 cm^−1^ was found for the angular vibration of C=O, and 1197.18 cm^−1^ was found for the angular vibration at C–O–C on the pyran ring and for C–OH stretching vibrations. EUPS exhibited a characteristic absorption peak at 793.15 cm^−1^, indicating that the polysaccharide contained a β-type glycosidic bond in its structure. The detailed structures of a polysaccharide are prerequisites for investigating its underlying mechanism and structure-activity relationships [23]. Studies indicate that EUPSs have a characteristic absorption band in the range of 1000–1200 cm^−1^. This absorption band is dominated by ring vibrations that overlap with the C–O–C glycosidic band vibrations and C–OH stretching vibrations in the side groups. The absorption peaks at ~1118.54, 1153.24, and 1263.77 cm^−1^ indicate that EUPSs are pyranose sugars. Furthermore, the absorption peak at 890.79 cm^−1^ suggests that the polysaccharides contain a β-type glycosidic bond in their structures [24].

#### 2.4.4. Analysis of Monosaccharide Components

**Figure 4:**
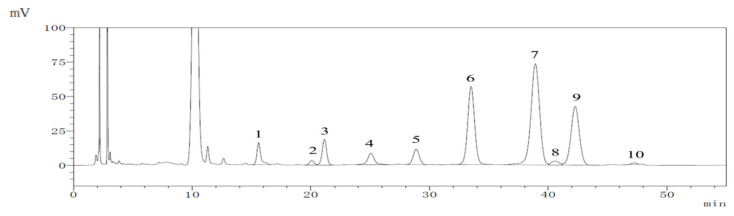
HPLC spectra of EUPS: 1. mannose, 2. ribose, 3. rhamnose, 4. glucuronic acid, 5. galacturonic acid, 6. glucose, 7. galactose, 8. xylose, 9. arabinose, and 10. fucose.

Usually, treatment with acid and high temperature leads to the partial degradation of carbohydrates to form byproducts. Among them, monosaccharides originating from polysaccharides can be degraded into several byproducts, such as furfural, 5-hydroxymethylfurfural, and carboxylic acids [25]. After high-performance liquid chromatography analysis, the monosaccharide components of the polysaccharide obtained are shown in Figure 4. Galactose is a dominant sugar component in EUPS, with a relative mass of 37.5% of the total sugar composition. Glucose (23.1%) and arabinose (20.9%) were observed as minor constituents in all polysaccharides, whereas rhamnose (4.9%), galacturonic acid (4.0%), mannose (3.7%), glucuronic acid (3.3%), xylose (1.31%), ribose (0.9%), and fucose (0.3%) were present in small amounts. This finding is consistent with the results of previous studies that reported that the polysaccharides mainly comprise mannose, rhamnose, galacturonic acid, glucose, galactose, xylose, and arabinose at mass percentages of 1.29%, 2.18%, 3.15%, 82.7%, 0.79%, 2.02%, and 1.99%, respectively [26].

#### 2.4.5. Determination of Molecular Weight of EUPS

The molecular weight distribution of the EUPS was determined via gel permeation chromatography. The results show that the weight-average molecular weight (M_w_) was ~538.0 kDa, the number-average molecular weight (M_n_) was ~101.3 kDa, and the polydispersity (M_w_/M_n_) was 5.3. This was demonstrated in numerous studies, where the molecular weight of purified EUPS was 630–251,000 Da.

#### 2.4.6. Determination of Particle Size of EUPS

**Figure 5:**
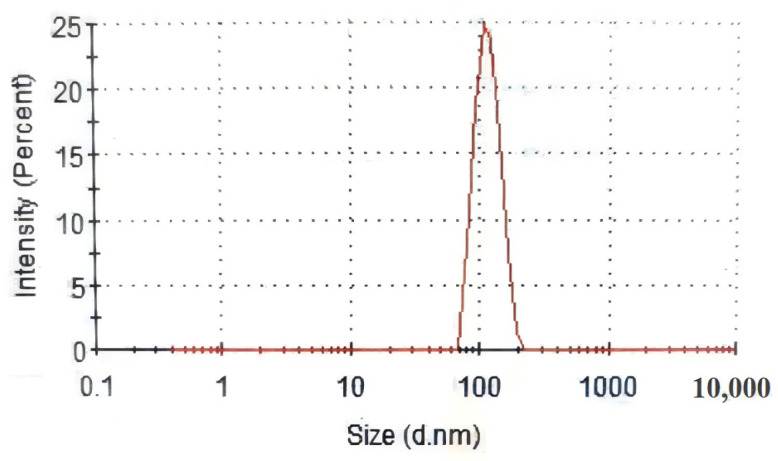
Particle size distribution of EUPS.

The particle sizes of the EUPSs were determined using a laser particle size analyzer. The results obtained are shown in Figure 5. It was determined that the particle size distribution of EUPS was concentrated at ~140.18 nm, and the particle size was relatively uniform.

### 2.5. Antioxidant Activities

#### 2.5.1. DPPH Radical-Scavenging Activity

**Figure 6:**
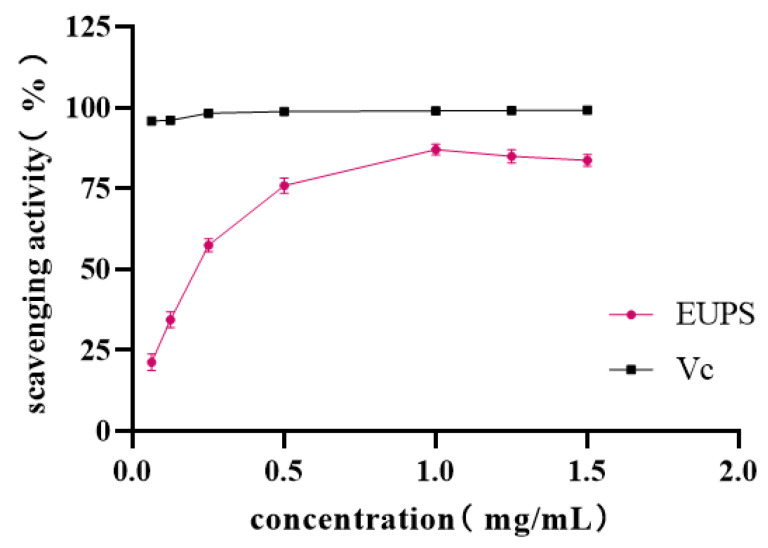
Scavenging ability of EUPS and Vc for DPPH radicals.

The scavenging capacity of EUPS on DPPH radicals was expressed by the scavenging rate. The higher the scavenging rate, the stronger the antioxidant effect of the extracts. The scavenging ability of the EUPS to DPPH radicals is shown in Figure 6. Within the concentration of 1.0 mg/mL, the scavenging ability to DPPH radicals rapidly improved with increasing concentration. When the concentration was 1.0 mg/mL, the scavenging ability reached 87.05%. Within the concentration between 1.0 and 1.5 mg/mL, with increasing concentration, the scavenging ability of the DPPH radicals decreased slowly. The results indicate that the polysaccharides from *E. ulmoides* exhibit a certain scavenging activity against DPPH radicals. These results agree with those of Liang et al., who reported that the DPPH free radical IC_50_ of *Dendrobium* polysaccharide was 0.227 mg/mL, indicating that *Dendrobium* polysaccharide exhibited high antioxidant activity [27]. Fakhfakhf’s research also showed that the IC_50_ of the DPPH free radical of mallow polysaccharide was 1.25 mg/mL [28].

#### 2.5.2. ABTS Radical-Scavenging Activity

**Figure 7:**
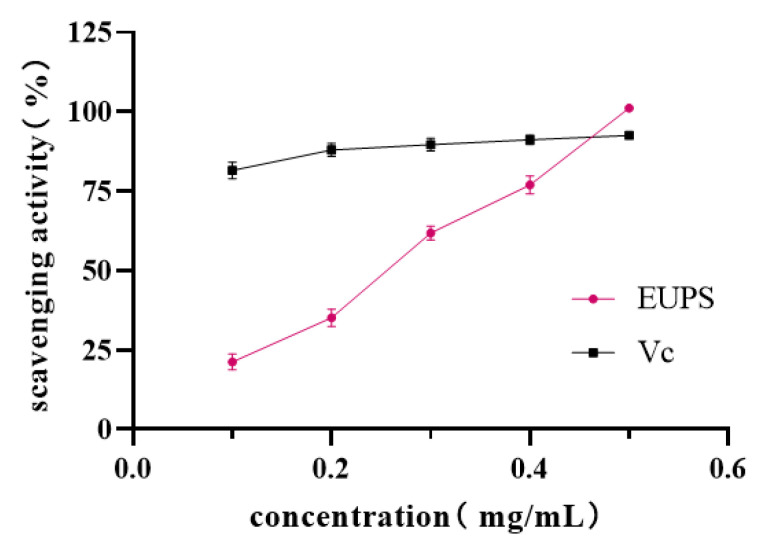
Scavenging ability of EUPS and Vc for ABTS radicals.

To further validate the antioxidant ability of EUPSs, ABTS radical cation-scavenging activity was assessed. ABTS is oxidized by potassium persulfate to generate ABTS+. Antioxidants can react with ABTS+ to generate colorless reductive ABTS. The ability of antioxidants to scavenge the ABTS radical cation can be evaluated by detecting the attenuation of the absorbance after mixing samples and ABTS+ at 734 nm [29]. Figure 7 shows that the scavenging ability of EUPS on ABTS radicals and polysaccharide concentration exhibited a dose-effect relationship within a certain concentration range. With an increase in the concentration of polysaccharides, the scavenging ability of polysaccharides on ABTS radicals gradually increased. When the concentration was 0.5 mg/mL, the scavenging ability of the ABTS radicals was stronger and the scavenging rate reached 101.17%. These results show that EUPS plays an antioxidant role via the electron transfer mechanism. In addition, the imbalance in the production and consumption of ROS leads to oxidative stress, and normalizing the production of ROS can exert antioxidant effects. Polysaccharides contain a hemiacetal hydroxyl group; hence, they can react with active oxygen and alleviate oxidative stress [30].

#### 2.5.3. Hydroxyl Radical-Scavenging Activity

**Figure 8:**
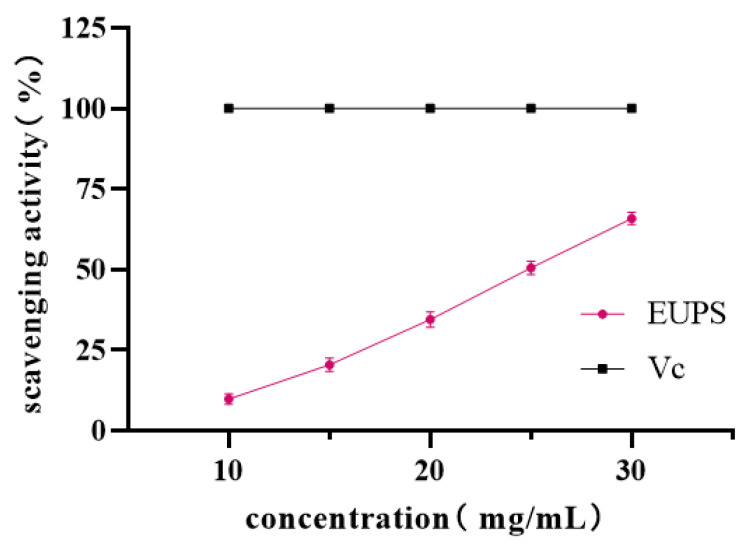
Scavenging ability of EUPS and Vc to hydroxyl radicals.

The scavenging ability of the EUPS to hydroxyl radicals is shown in Figure 8. Similar to scavenging ABTS radicals, the polysaccharide concentration exhibited a dose-effect relationship with hydroxyl radicals within a certain concentration range. With an increase in concentration, the scavenging ability for hydroxyl radicals significantly improved. When the concentration was 30 mg/mL, the scavenging ability of the hydroxyl radicals was stronger, and the scavenging rate reached 65.92%. This finding was consistent with a previous finding that showed that the IC_50_ of ·OH free radical clearance of Ginkgo polysaccharide was 0.482 mg/mL [31].

#### 2.5.4. Reduction Ability

**Figure 9:**
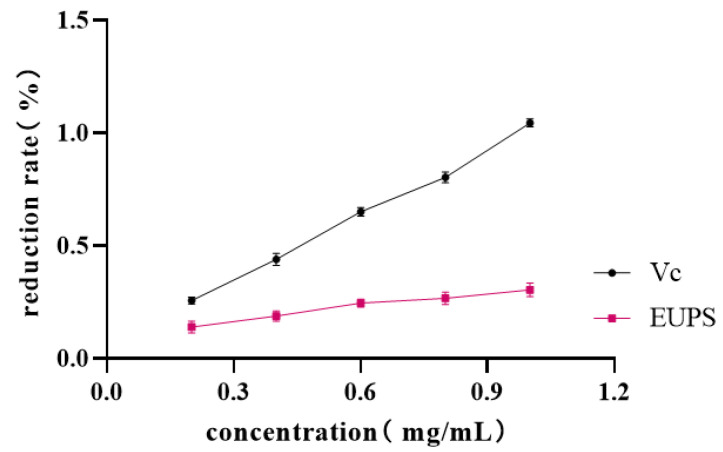
Reduction ability of EUPS and Vc.

The reductive capacity of the EUPS and its derivatives are shown in Figure 9. With an increase in concentration, the reduction ability significantly improved. The absorbance of EUPS was 1.044 at a concentration of 1.0 mg/mL. This shows that the EUPS exhibit a certain reducing ability and is suitable for use as a reducing agent. Increasing evidence has shown that the monosaccharide composition of polysaccharides and the ratios of monosaccharides could exert an effect on their antioxidant capacities [32,33,34]. Monosaccharides with active hydroxyl groups can provide hydrogen or electrons with free radicals, thereby forming stable free radicals and terminating free radical chain reactions. The main monosaccharide of the EUPS is galactose, which has a good active hydroxyl group. It is speculated that this may be the reason why EUPS exhibits good total reducing power [35,36]. Radical-scavenging activities were significantly correlated with mannose and glucose contents. In addition, the higher the mannose content, the better the radical scavenging ability [37]. Shang et al. reported that the antioxidant capacity of polysaccharides was related to the content of glucuronic acid, indicating that the better total reducing power of EUPS might be because of the higher content of glucuronic acid [38].

#### 2.5.5. Antioxidant Indices in the Serum of Mice

**Figure 10:**
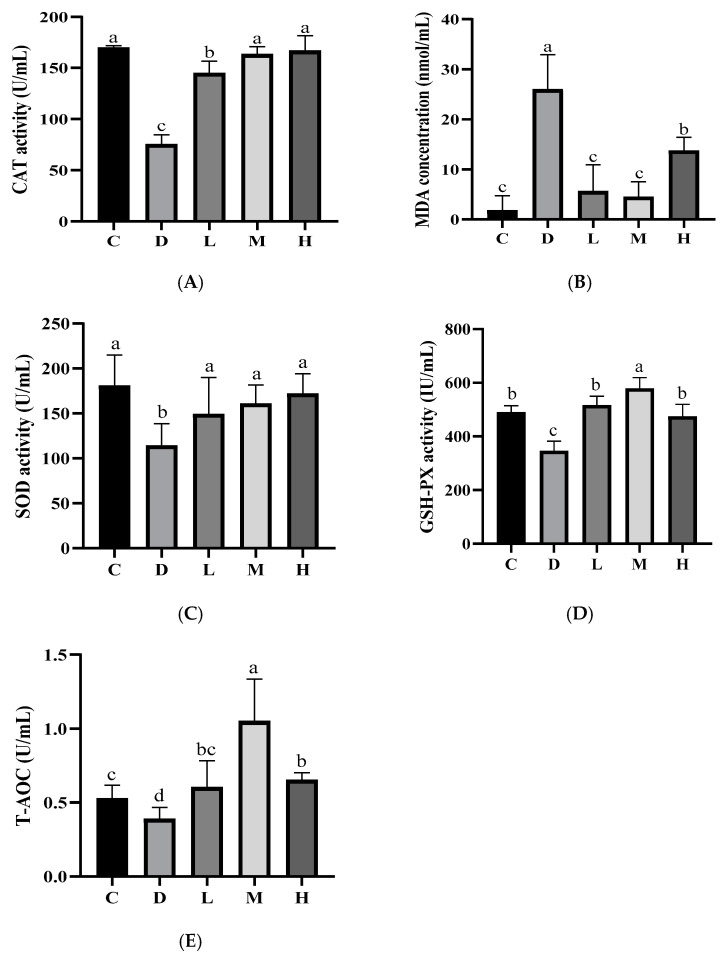
Effect of EUPS on antioxidant activities in the serum of mice. (**A**) Serum CAT activity; (**B**) serum MDA activity; (**C**) serum SOD activity; (**D**) serum GSH-Px activity; (**E**) serum T-AOC activity. The data are expressed as the means ± SEM (*n* = 10); the different letters indicate the statistically significant differences in all of the treatment groups (*p* < 0.05).

Serum index is an important parameter reflecting the metabolism of body activity [39]. As shown in Figure 10, compared with Group C, Group D showed a decreased activity in CAT, SOD, GSH-Px, and T-AOC (*p* < 0.05), whereas the MDA content was increased (*p* < 0.05). The accumulation of MDA content can not only reflect the intensity of lipid peroxidation, but also reflect the degree of oxidative damage [40]. EUPS groups induced an increase in the activities of SOD and T-AOC (*p* < 0.05), but the MDA levels decreased (*p* < 0.05). SOD activity indirectly reflects the body’s ability to scavenge free radicals. T-AOC is a comprehensive index to measure the antioxidant capacity of all antioxidant substances and antioxidant enzymes in an animal’s body [41]. Group M and Group H increased the activities of SOD and T-AOC compared with Group D. Among the three EUPS groups, CAT activity increased in Group M and Group H (*p* < 0.05). This shows that EUPS can increase the body‘s antioxidant capacity, enhance its ability to scavenge free radicals, and reduce cell damage in mice. The antioxidant capacity of mice in the EUPS groups was significantly higher than that in Group D. With the increase in EUPS, the GSH-PX and T-AOC in the body increased first and then decreased gradually, and the MDA level decreased first and then increased. This may be due to the increase in cell osmotic pressure caused by a high concentration of EUPS.

### 2.6. RNA Extraction, Reverse Transcription, and Fluorescence Quantitative PCR

**Figure 11:**
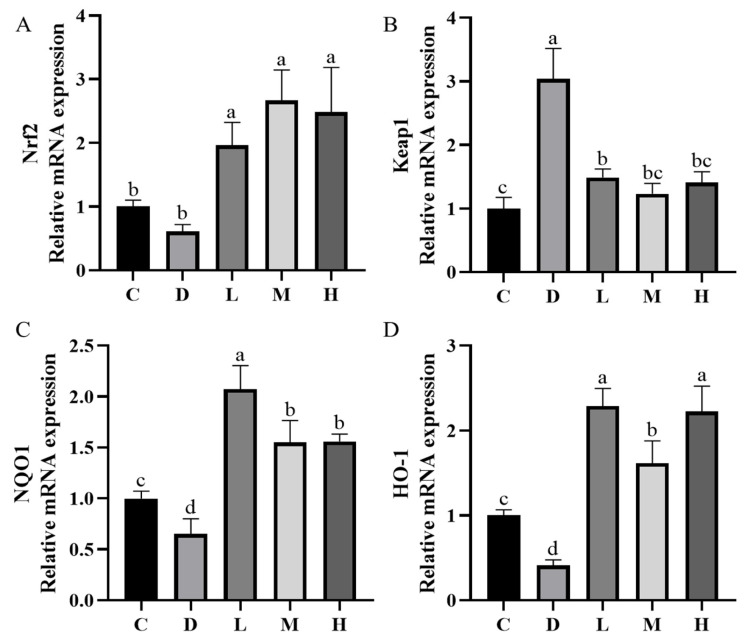
Expression levels of Nrf2 genes (**A**), Keap1genes (**B**), NQO1 genes (**C**), and HO-1 genes (**D**) in mice liver. The data are expressed as the means ± SEM (*n* = 10); the different letters indicate the statistically significant differences in all of the treatment groups (*p* < 0.05).

As shown in Figure 11, the expression of NF-E2—related factor 2 (Nrf2) in the liver increased (*p* < 0.05) in the EUPS groups compared with Group C and Group D. The mice in Group D and Group L exhibited higher (*p* < 0.05) mRNA abundance of Kelch-like ECH-associated protein l (Keap1), but lower (*p* < 0.05) NAD(P)H:quinoneoxidoreductase 1 (NQO1) and heme oxygenase 1 (HO-1) mRNA expression in the liver in Group D compared to Group C. The administration of EUPS inhibited a diquat-induced decrease in NQO1 and HO-1 mRNA expression in the liver of mice. Among the three EUPS groups, the expression of NQO1 in the liver decreased in Group M and Group H (*p* < 0.05), and the expression of HO-1 in the liver decreased in Group M (*p* < 0.05).

### 2.7. Determination of Intestinal Microorganisms

#### 2.7.1. Composition and Abundance of Gut Microflora

**Figure 12:**
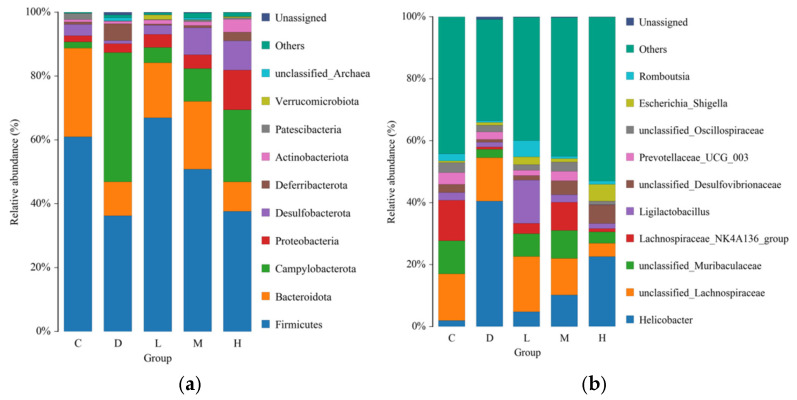
Effects of EUPS on species composition. (**a**) Histogram of relative abundance at the phylum level. (**b**) Histogram of relative abundance at the genus level.

At the phylum level, ten bacterial phyla were annotated (Figure 12a): Firmicutes, Bacteroidetes, Campylobacterota, Proteobacteria, and the four main bacteria (Desulfobacterota, Dferribacterota, Actinobacteria, Patescibacteria). Diquat treatment decreased the abundance of dominant Firmicutes by 40.55% compared with Group C (*p* < 0.05). Compared with Group D, Firmicutes were significantly increased by 84.58% in Group L, by 40.25% in Group M, and by 3.87% in Group H. The ratio of Firmicutes to Bacteroidetes in EUPS groups was increased compared to Group C. The ratio of Firmicutes to Bacteroidetes is recognized as an important indicator of intestinal flora dysfunction [42]. Firmicutes include many beneficial bacteria that can metabolize short-chain fatty acid salts, such as acetate and lactate, thereby regulating the proportion of symbiotic bacteria in the gut, reducing intestinal inflammation, and regulating immune balance [43].

The top ten bacteria generally at the genus level are shown in Figure 12b. After diquat treatment, the abundance levels of *Helicobacter* increased compared with that in Group C. However, the abundance of *Helicobacter* significantly decreased by 88.18%, 84.69%, and 44.13% (*p* < 0.05) after treatment with low, medium, and high doses of EUPSs, respectively. In addition, EUPS treatment increased the abundance of Lachnospiraceae, *Ligilactobacillus*, and *Romboutsia* to different degrees. Lachnospiraceae participate in the metabolism of most carbohydrates and produce acetic acid, butyric acid, and other short-chain fatty acids via fermentation. Therefore, it regulates obesity and related glucose and lipid metabolism disorders; Lachnospiraceae is a beneficial bacteria [44]. The antioxidant activity of *Ligilactobacillus* can alleviate oxidative stress reactions in the body. In addition, *Romboutsia* can produce short-chain fatty acids, such as butyric acid, which affect colon movement. Therefore, *Romboutsia* has certain anti-inflammatory properties [45,46]. Thus, EUPS can improve the dysbiosis of the gut microbiota.

#### 2.7.2. Alpha Diversity Analysis

**Figure 13:**
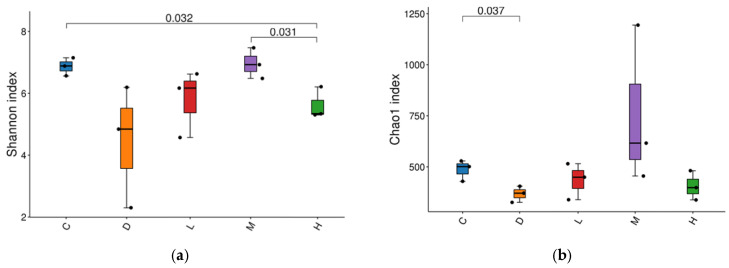
Alpha diversity of gut microbiota. (**a**) Chao1 index. (**b**) Shannon index. Each dot in the graph represents a sample, and dots of different colors indicate different groups.

To show the alpha diversity characteristic of the data distribution, a boxplot of Chao 1 and Shannon indices was used to investigate the diversity of the gut flora community. As shown in Figure 13, the alpha diversity of the gut microbiota in Group C was similar to that in the EUPS groups, indicating that treatment with EUPS could improve the richness and diversity of gut microbiota after diquat treatment. The Chao1 index was used to evaluate the number of intestinal bacterial communities, whereas Shannon reflects the diversity [47]. As shown in Figure 13a, compared with Group C, the Chao1 index of Group D was significantly reduced, but there was no significant difference between Groups C and L, Group M, and Group H. These results indicate that EUPS could reduce the number of species in the community caused by oxidative stress injury in mice. Figure 13b shows that the Shannon index of Group H was significantly lower than that of Group C, whereas the difference between Group C and Groups D, L, and M was not significant, and the Shannon index of Group H was significantly lower than that of Group M. These results show that the cecal microbial diversity of mice in Group H was significantly higher than that in the other four groups, indicating that EUPS can improve intestinal microbial diversity.

#### 2.7.3. Beta Diversity Analysis

Beta diversity is a measure of microbial community composition and evaluates differences among microbial communities. Herein, we determined the beta diversity of each group using unweighted pair-group method with arithmetic means (UPGMA), principal coordinate analysis (PCoA), and nonmetric multidimensional scaling (NMDS).

**Figure 14:**
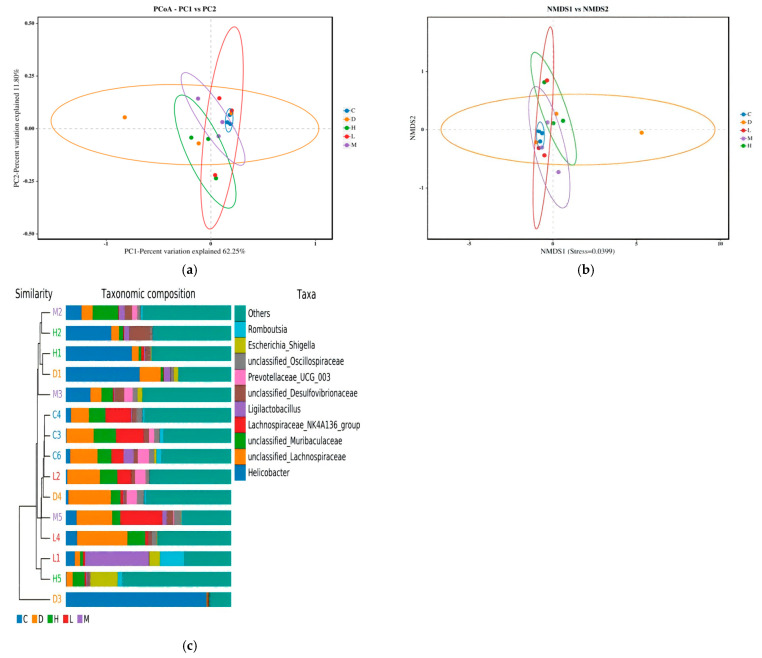
Beta diversity analysis. (**a**) PCoA analysis. (**b**) NMDS analysis. (**c**) UPGMA.

The beta diversity analysis is shown in Figure 14. The model mice had significantly different gut flora compositions from the control mice (Figure 14a,b), and the composition of bacteria within the groups was similar (Figure 14c). However, after treatment, the gut microbiota dysbiosis improved in the EUPS groups.

## 3. Materials and Methods

### 3.1. Materials

*E. ulmoides* leaves were collected from Henan Jinduzhong Agricultural Technology Co., Ltd., in Xuchang of Henan province, China. The leaves of *E. ulmoides* were picked in July, and the tree age was 2 years old. The leaves of *E. ulmoides* were washed with running water, placed in an electrothermal constant-temperature blast drying oven (model DH6-924385-Ⅲ, Shanghai Xinmiao Medical Treatment Apparatus Manufacturing Co., Ltd., Shanghai, China) and dried at 40 °C to constant weight. The dried leaves were ground into a fine powder and then passed through a 60-mesh sieve.

### 3.2. Extraction and Purification of Polysaccharides

The powder of the pretreated dried leaves was defatted with petroleum ether and refluxed with 95% ethanol to remove colored components or small molecular impurities. Then, 3.000 g of ground leaf samples were extracted using an ultrasound cleaner (model FRQ-1004T, Frant Ultrasonic Technology Co., Ltd., Shenzhen, China). The leaf extract was centrifuged at 3000 r/min for 10 min. Then, four times the volume of 95% ethanol was added to the supernatant. The concentrated extract was precipitated via ethanol overnight and then centrifuged (model Thermo Scientific SL8, Thermo Fisher Scientific, Waltham, MA, USA) for 10 min at 3000 r/min, following which the supernatant was discarded and the precipitates were obtained. The precipitates were solubilized in distilled water, and the mixed solution was immersed in equal volume of 6% trichloroacetic acid overnight at 4 °C, centrifuged for 10 min at 3000 r/min, and the precipitates were discarded. D101 macroporous resin was added to the supernatant and then decolorized in a 60 °C water bath for 30 min to remove colored ingredients. The extract was collected via filtration, concentrated using a rotary evaporator (model RE-3000A, Shanghai Zhiyi Technology Co., Ltd., Shanghai, China) at 70 °C under vacuum, precipitated using ethanol overnight at 4 °C, and centrifuged for 10 min at 3000 r/min. The precipitates were solubilized in distilled water, deproteinized in a dialysis bag for 72 h, precipitated via ethanol, low-temperature vacuum dehydration treatment was carried out at −55 °C, and EUPS were obtained [48].

### 3.3. Response Surface Method

According to the principles of the Box-Behnken central combination design (BBD), response surface analysis tests were designed for 17 experimental sites with three factors and three levels, including the ratio of water to raw material (A), extraction temperature (°C, B), and extraction time (h, C). The extraction yield (%) of EUPS was considered to be the response. SAS 9.2 software was used to determine the factor level of the response surface analysis. Theoretical optimal conditions were obtained from the test results, and further tests were conducted to verify the effectiveness of the model [49].

### 3.4. Structure of EUPS

#### 3.4.1. Physicochemical Property Analysis

The physicochemical properties of EUPSs were determined via color and texture observations. The contents of soluble starch reducing sugar, protein, and total polyphenol were determined via an iodination test, the Folin-Ciocalteu method, Coomassie brilliant blue reaction, and FeCl_3_ test, respectively [50].

#### 3.4.2. Uronic Acid Analysis

Uronic acid contents were determined via the carbazole-sulfuric acid method. EUPS (1.0 mg) and galacturonic acid were first dissolved in 1.0 mL of water. Sulfuric acid containing sodium tetraborate was thoroughly mixed with the sample solution in a test tube and incubated in a water bath at a temperature of 100 °C for 20 min. The mixture was cooled to room temperature, 0.2 mL of m-hydroxydiphenyl (0.15%) was added, and the resulting solution was completely mixed for 2 h. The absorbance of the measured sample was read at 523 nm [51].

#### 3.4.3. Fourier-Transform Infrared Spectroscopy Analysis

Purified EUPS was dried and ground with KBr powder and pressed into pellets. Fourier-transform infrared (FT-IR) spectroscopy was performed to investigate the FT-IR spectrum, which was scanned in a range from 4000 to 400 cm^−1^ using an FT-IR spectroscope (model FTIR-650S, Tianjin Gangdong SCI&TECH. Co., Ltd., Tianjin, China).

#### 3.4.4. Monosaccharide Composition Analysis

The monosaccharide composition of EUPS was determined using lipid chromatography (LC, Shimadzu, LC-20AD, Kyoto, Japan) on a Xtimate C_18_ column (4.6 mm × 200 mm). The column was eluted using 0.05 M KH_2_PO_4_ and acetonitrile as a mobile phase at a flow rate of 1.0 mL/min and injection volume of 20 μL [52].

#### 3.4.5. Molecular Weight Determination of EUPS

The average molecular weight of EUPS was determined using high-performance gel permeation chromatography (Thermo U3000, Waltham, MA, USA) with Ohpak SB-805 HQ and Ohpak SB-804 HQ columns (300 mm × 8 mm). The column was eluted using 0.02% NaN_3_ and 0.1 M NaNO_3_ as the mobile phase at a flow rate of 0.5 mL/min and an injection volume of 100 μL [53].

#### 3.4.6. Particle Size

The samples were dissolved in deionized water to prepare 1 mg/mL EUPS solution, and the particle size of the EUPS was detected using a Zeta potential particle size analyzer (model Zetasizer Ultra, Malvern Panalytical, Malvern, UK) [54].

### 3.5. Antioxidant Activity of EUPS

#### 3.5.1. Assay of DPPH Radical-Scavenging Activity

First, 1.0 mL EUPS samples at different concentrations (0.0625, 0.125, 0.25, 0.5, 1.0, 1.25, and 1.5 mg/mL) were incubated with 3.0 mL methanol solution of DPPH (Shanghai Acmec Biochemical Technology Co., Ltd., Shnaghai, China) at 25 °C for 30 min in the dark. The absorbance was recorded at 517 nm as A_1_. Anhydrous ethanol instead of DPPH solution was used to determine the absorbance as A_2_ using the above method, and the absorbance measured by the above method using distilled water instead of the sample solution was A_0_. At the same time, VC (Vitamin C) was used as a positive control to replace the sample solution to repeat the above steps [55].

#### 3.5.2. Assay of ABTS Radical-Scavenging Activity

First, 100 μL EUPS sample solution at different concentrations (0.1, 0.2, 0.3, 0.4, and 0.5 mg/mL) was taken into different test tubes, to which 3 mL of ABTS working solution (Shanghai Macklin Biochemical Technology Co., Ltd., Shanghai, China) was added. The reaction was shaken well and left for 6 min in the dark. The absorbance was recorded as A_1_ at 734 nm, with distilled water instead of ABTS solution according to the above method to determine the absorbance as A_2_, and with distilled water instead of sample solution according to the above method to determine the absorbance as A_0_. At the same time, VC (Vitamin C) was used as a positive control to replace the sample solution to repeat the above steps [56].

#### 3.5.3. Assay of Hydroxyl Radical-Scavenging Activity

First, 1.0 mL of EUPS sample solution at different concentrations (10, 15, 20, 25, and 30 mg/mL) was taken into a different test tube. Then, 1 mL FeSO_4_ solution (0.15 mmol/L), 0.4 mL salicylic acid solution (2 mmol/L), 1.0 mL H_2_O_2_ (4.0 mmol/L), and 0.4 mL distilled water were added. The reaction mixture was incubated for 1 h at 37 °C. The absorbance was recorded as A_1_ at 510 nm, with distilled water instead of salicylic acid solution according to the above method to determine the absorbance as A_2_, and with distilled water instead of sample solution according to the above method to determine the absorbance as A_0_. At the same time, VC (Vitamin C) was used as a positive control to replace the sample solution to repeat the above steps [57].

The DPPH radical-scavenging effect, the ABTS radical-scavenging capacity, and the hydroxyl radical-scavenging effect were calculated using the following equation:

$$Scavenged\;hydroxyl\;(\%)=[1-(A_{1}-A_{2})/A_{0}]\times 100\%,$$

#### 3.5.4. Assay of Reduction Ability

First, 1.0 mL of EUPS sample solution at different concentrations (0.2, 0.4, 0.6, 0.8, and 1.0 mg/mL) was taken into different test tubes, to which 0.2 mL of phosphate buffer and 0.5 mL of K_3_Fe(CN)_6_ solution were added. The reaction was conducted at 50 °C for 20 min, and then cooled rapidly. Then, 2.5 mL of 10% Cl_3_CCOOH solution was added and centrifuged for 10 min at 3000 r/min; the supernatant was obtained via filtration. Next, 1.5 mL of the supernatant was absorbed, 0.2 mL of 0.1% FeCl_3_ solution and 3 mL of distilled water were added, the reaction was shaken well and left to stand for 5 min, and then the absorbance was recorded as A_1_ at 700 nm, with distilled water instead of the K_3_Fe(CN)_6_ solution according to the above method to determine the absorbance as A_2_, and with distilled water instead of sample solution according to the above method to determine the absorbance as A_0_. At the same time, VC (Vitamin C) was used as a positive control to replace the sample solution to repeat the above steps [58]. The reduction ability was calculated using the following equation:

$$\mathrm{Reduction}\;\mathrm{ability}\;(\%)=(A_{1}-A_{0}-A_{2})\times 100\%,$$

### 3.6. Animals and Treatment

Thirty male four-week-old Kunming mice (20.95 ± 1.71 g), which were obtained from Huaxing experimental animal farm, were used in this study. The animal study protocol was approved by the Ethics Committee of Henan University of Technology (HAUTETHI-2022-0723).

Experimental mice were housed in a pathogen-free environment with free access to standard chow and water for 7 d in an automatically controlled 12 h light/dark cycle with a temperature of 22 °C ± 2 °C. Experimental mice were randomly divided into five groups (n = 10), and each received a daily gavage of the same volume solution, as follows: saline (Group C), diquat (Group D), 100 mg/kg EUPS (Group L), 200 mg/kg EUPS (Group M), or 400 mg/kg EUPS (Group H) for 14 consecutive days. Group C was injected intraperitoneally with saline, and the other four treatment groups were injected intraperitoneally with diquat (25 mg/kg BW) at 9 pm on day 14. At the end of the experiment, the mice were euthanized and their blood samples were collected from the orbital sinus and centrifuged at 8000 rpm for 10 min to obtain the serum, which was stored at 4 °C for further investigation. The liver and cecum contents were collected rapidly. All tissue samples were divided into two parts: one was frozen in liquid nitrogen and then stored at −80 °C for gene expression and biochemical analyses, and the other one was fixed in 4% paraformaldehyde for subsequent histopathological examination. The samples of stool and cecal contents were collected in plastic tubes and immediately frozen at −80 °C for further analysis.

#### 3.6.1. Determination of Biochemical Indices

CAT, SOD, GSH-Px, total antioxidant capacity (T-AOC) activities, and malondialdehyde (MDA) levels in the serum were determined by strictly following the commercial kit instructions (Nanjing Jiancheng Bioengineering Institute, Nanjing, China) [59].

#### 3.6.2. RNA Extraction and Quantitative Real-Time Polymerase Chain Reaction

Total RNA from the liver was extracted using TRIzol Reagent following the manufacturer’s instructions (Invitrogen; Thermo Fisher Scientific, Inc. Waltham, MA, USA). RNAs were reverse transcribed into cDNAs following the manufacturer’s instructions (Biomed, RA101-12, Waltham, MA, USA). Real-time PCR was performed using PrimeScript SYBR^®^ Premix Ex Taq™ II (Tli RNaseH Plus) in a Step OnePlus System (Biosystems, Foster City, CA, USA). The results of mRNA expression were calculated using the 2^−ΔΔCt^ method and normalized to *β-actin* expression. The primer sequences used for gene expression detection in this study are listed in Table 3.

#### 3.6.3. Gut Microbiota Analysis

Fresh digesta isolated from the cecum were immediately sent to Qingke Biotechnology Co., Ltd. (Beijing, China) for gut microbiota analysis. The entire region of the 16S rRNA gene was augmented via PCR using specific primers (F: ACTCCTACGGGAGGCAGCA; R: GGACTACHVGGGTWTCTAAT), followed by mixing and purification of the PCR products and construction of a DNA library [60,61].

#### 3.6.4. Statistical Analysis

Data are shown as mean ± standard deviation or mean ± standard error of the mean. The statistical analysis was performed via one-way analyses of variance (ANOVA) using SPSS (v.21.0, SPSS, Inc., Chicago, IL, USA).

## 4. Conclusions

Herein, the conditions for extracting EUPS were optimized using response surface methodology (RMS) with a Box-Behnken design (BBD). The optimal conditions were as follows: a ratio of water to raw materials of 22 mL/g, extraction temperature of 63 °C, and extraction time of 1 h. Under these conditions, the extraction yield was 2.60%. The separation and purification method of EUPS was studied, and its components were analyzed. Only an elution peak was detected using column chromatography, indicating that the EUPS is homogeneous with a number-average molecular weight of 101.3 kDa. In vitro experiments revealed that EUPS exhibited a good scavenging effect on DPPH, ABTS, and hydroxyl radicals, and a significant dose-effect relationship. The results show that EUPSs are potential natural antioxidants. Subsequently, different doses of EUPSs were orally administered in mice models of oxidative damage. Results showed that EUPSs improved oxidative stress by upregulating Nrf2, HO-1, and NQO1 expression, downregulating Keap1 expression, and modulating the intestinal flora structure, with a similar effect in liver tissue.

## Figures and Tables

**Table 1:** Experimental results of the response surface methodology ^a^.

Run	Ratio of Water to Materials A (mL/g)	Extraction Temperature B (°C)	Extraction Time C (h)	Yield (%) (*w*/*w* Dry Weight)
1	−1 (15:1)	−1 (40)	0 (1)	2.18
2	1 (25:1)	−1 (40)	0 (1)	2.35
3	−1 (15:1)	1 (80)	0 (1)	2.32
4	1 (25:1)	1 (80)	0 (1)	2.45
5	−1 (15:1)	0 (60)	−1 (0.5)	2.35
6	1 (25:1)	0 (60)	−1 (0.5)	2.44
7	−1 (15:1)	0 (60)	1 (1.5)	2.33
8	1 (25:1)	0 (60)	1 (1.5)	2.49
9	0 (20:1)	−1 (40)	−1 (0.5)	2.21
10	0 (20:1)	1 (80)	−1 (0.5)	2.34
11	0 (20:1)	−1 (40)	1 (1.5)	2.29
12	0 (20:1)	1 (80)	1 (1.5)	2.41
13	0 (20:1)	0 (60)	0 (1)	2.57
14	0 (20:1)	0 (60)	0 (1)	2.6
15	0 (20:1)	0 (60)	0 (1)	2.59
16	0 (20:1)	0 (60)	0 (1)	2.55
17	0 (20:1)	0 (60)	0 (1)	2.62

^a^ Mean of triplicate determination.

**Table 2:** ANOVA for response surface quadratic model analysis of variance.

Source	Sum of Squares	Degree Freedom	Means of Squares	*F* Value	*p* Value	Significant
Model	0.29	9	0.033	5.38	<0.0001	**
A-A	0.038	1	0.038	23.16	0.0002	**
B-B	0.03	1	0.03	2.2	0.0003	**
C-C	4.050 × 10^−3^	1	4.050 × 10^−3^	1.17	0.0496	*
AB	4.000 × 10^−4^	1	4.000 × 10^−4^	5.34	0.4805	
AC	1.225 × 10^−3^	1	1.225 × 10^−3^	1.77	0.2336	
BC	2.500 × 10^−5^	1	2.500 × 10^−5^	4.87	0.8575	
A^2^	0.031	1	0.031	1.83	0.0003	**
B^2^	0.13	1	0.13	2.94	<0.0001	**
C^2^	0.040	1	0.040	4.09	0.0001	**
Residual	5.045 × 10^−3^	7	7.207 × 10^−4^			
Lack of fit	2.125 × 10^−3^	3	7.083 × 10^−4^	0.97	0.4893	
Pure error	2.920 × 10^−3^	4	7.300 × 10^−4^			
Cor total	0.3	16				

Significant differences are expressed as * *p* < 0.05 and ** *p* < 0.01.

**Table 3:** Primers sequences for RT-PCR.

Gene	Nucleotide Sequence of Primers (5′–3′)	PubMed No	Product Length
Nrf2	F: ACCTCTGCTGCAAGTAGCCT R: TGGGCAACCATCACTCTGCT	NM_001399226.1	118
Keap1	F: GCCCCGGGACTCTTATTGTG R: TTAGGGGCCCCGCCAT	NM_001110305.1	101
HO-1	F: GCTAGCCTGGTGCAAGATACT R: AAGCTGAGAGTGAGGACCCA	NM_010442.2	110
NQO1	F: CTCTGGCCGATTCAGAGTGG R: CTCCCAGACGGTTTCCAGAC	NM_008706.5	147
β-actin	F: CATTGCTGACAGGATGCAGA R: CTGCTGGAAGGTGGACAGTGA	NM_007393.5	118

## Data Availability

The data generated from the study is clearly presented and discussed in the manuscript.
